# Albino T-DNA tomato mutant reveals a key function of 1-deoxy-D-xylulose-5-phosphate synthase (DXS1) in plant development and survival

**DOI:** 10.1038/srep45333

**Published:** 2017-03-28

**Authors:** Manuel García-Alcázar, Estela Giménez, Benito Pineda, Carmen Capel, Begoña García-Sogo, Sibilla Sánchez, Fernando J. Yuste-Lisbona, Trinidad Angosto, Juan Capel, Vicente Moreno, Rafael Lozano

**Affiliations:** 1 Centro de Investigación en Biotecnología Agroalimentaria (BITAL), Universidad de Almería, 04120, Almería, Spain; 2 Instituto de Biología Molecular y Celular de Plantas (UPV-CSIC), Universidad Politécnica de Valencia, 46022, Valencia, Spain

## Abstract

Photosynthetic activity is indispensable for plant growth and survival and it depends on the synthesis of plastidial isoprenoids as chlorophylls and carotenoids. In the non-mevalonate pathway (MEP), the 1-deoxy-D-xylulose-5-phosphate synthase 1 (DXS1) enzyme has been postulated to catalyze the rate-limiting step in the formation of plastidial isoprenoids. In tomato, the function of DXS1 has only been studied in fruits, and hence its functional relevance during plant development remains unknown. Here we report the characterization of the *wls-2297* tomato mutant, whose severe deficiency in chlorophylls and carotenoids promotes an albino phenotype. Additionally, growth of mutant seedlings was arrested without developing vegetative organs, which resulted in premature lethality. Gene cloning and silencing experiments revealed that the phenotype of *wls-2297* mutant was caused by 38.6 kb-deletion promoted by a single T-DNA insertion affecting the *DXS1* gene. This was corroborated by *in vivo* and molecular complementation assays, which allowed the rescue of mutant phenotype. Further characterization of tomato plants overexpressing *DXS1* and comparative expression analysis indicate that DXS1 may play other important roles besides to that proposed during fruit carotenoid biosynthesis. Taken together, these results demonstrate that DXS1 is essentially required for the development and survival of tomato plants.

Photosynthesis is a vital process for plant development and survival which is precisely regulated at several genetic, molecular and physiological levels during the plant growth cycle^1^. Photosynthetic pigments (chlorophylls and carotenoids) are a group of molecules that belong to a large class of compounds of different biochemical nature, the isoprenoids. Isoprenoids comprise a chemically and functionally wide heterogeneous group including more than 30,000 molecules^2^. In addition to photosynthesis, plants produce many isoprenoids whose functions are essential in developmental processes such as growth regulation (gibberellins, cytokinins, brassinosteroids and abscisic acid), defense mechanism (phytoalexins), membrane structure (sterols) and redox chemistry (plastoquinone, ubiquinone). Despite their diversity, all isoprenoids are formed from two common precursors, isopentenyl diphosphate (IPP) and its isomer dimethylallyl diphosphate (DMAPP)^3^. In higher plants, there are two distinct cellular compartmentalized pathways for the biosynthesis of IPP and DMAPP^4^,^5^,^6^,^7^: the cytosolic mevalonate (MVA) pathway, which is shared with fungi, yeast, and animals^8^,^9^, and the non-mevalonate pathway designated as the 2-*C*-methyl-D-erythritol-4-Phosphate (MEP) pathway, located in plastids and also occurring in eubacteria, green algae and protozoa^10^,^11^,^12^,^13^,^14^. The initial step of the MEP pathway, a transketolase-type condensation of pyruvate with D-glyceraldhyde-3-phosphate to form 1-deoxy-D-xylulose-5-phosphate (DXP), is catalyzed by DXP synthase (DXS)^12^. The cloning and characterization of the *DXS* gene was first described in *Escherichia coli*^15^. *DXS* homologous genes were subsequently cloned from several plant species, including peppermint^16^, pepper^17^, Arabidopsis^18^ and tomato^19^. Since first reported, the *DXS* gene has been studied in diverse plant species in order to gain deeper knowledge of the DXS family, and functional diversification has been suggested in this family due to the existence of at least three functionally specialized types of DXS, i.e. DXS1, DXS2 and DXS3^20^,^21^,^22^. Despite high amino acid sequence homology among DXS proteins, mainly between DXS1 and DXS2, not all *DXS*-like genes have the same expression pattern during plant development. DXS1 has been proposed to be specifically involved in the synthesis of essential isoprenoids (such as photosynthetic pigments and phytohormones) in chloroplasts^18^,^19^. In turn, DXS2 is thought to be located in non-photosynthetic plastids, involved in the synthesis of specific isoprenoids as secondary metabolites related to mycorrhiza^23^. In addition, it has been suggested that DXS3 might participate in the synthesis of essential isoprenoids required at low levels, such as phytohormones^20^.

Many isoprenoids, carotenoids among them, have gained importance as biotechnological tools as they show beneficial effects on human health^24^,^25^. Since DXS1 is the key enzyme that limits output from the MEP pathway in plants^19^,^26^,^27^, changes in *DXS1* expression may directly influence the content of photosynthetic pigments. Many experiments have therefore been carried out to enhance the level of some isoprenoids in model and crop species, mainly by the generation of *DXS* overexpression transgenic lines^28^,^29^. Indeed, constitutive expression of a bacterial *DXS* gene under the control of the CaMV 35S promoter in transgenic tomato lines did alter the isoprenoid end products in fruits, but not in leaves^28^. Similarly, the overexpression of *DXS1* in Arabidopsis^26^ and the constitutive expression of the soybean *DXS1* gene in tobacco transgenic plants^29^ produced increases in various isoprenoids, including total chlorophylls and carotenoids. However, results from *DXS1* overexpression experiments differed among plant species, including tomato, indicating a complex regulation of DXS activity^28^. Additionally, characterization of *DXS* mutants has shed light on the key function of DXS proteins in plant development^26^,^30^. In Arabidopsis, DXS1 plays an essential role in chloroplast development during leaf cell maturation^30^,^31^, while DXS2 seems to have no DXS activity, but it has acquired another unknown biochemical function^32^. In tomato, the DXS1 function is required for carotenoid biosynthesis during fruit ripening^19^, while tomato DXS2 participates in the biosynthesis of isoprenoids in trichomes^16^,^22^,^33^ and of secondary metabolites^16^. Such results suggest that differences in the biochemical roles of DXS proteins seem to be related with variable functions in plant development and growth.

In this work, we report the identification and molecular characterization of the tomato *white lethal seedling*-2297 (*wls*-2297) mutant, which was isolated from the screening of a T-DNA mutant collection generated by an enhancer trapping gene construct. Seedlings of the *wls-2297* mutant showed albino phenotype and were unable to develop any true leaves from the shoot apical meristem; as a consequence, albino seedlings died after reaching the fully expanded cotyledon stage. Expression and functional analyses demonstrated that these phenothypic alterations were caused by the loss-of-function of the *DXS1* gene, indicating that, besides its role in fruit carotenoid biosynthesis, DXS1 activity is required at early stages of plant development and determines plant survival.

## Results

### The *wls-2297* mutant showed significant alterations in pigment content and plant development

**Figure 1:**
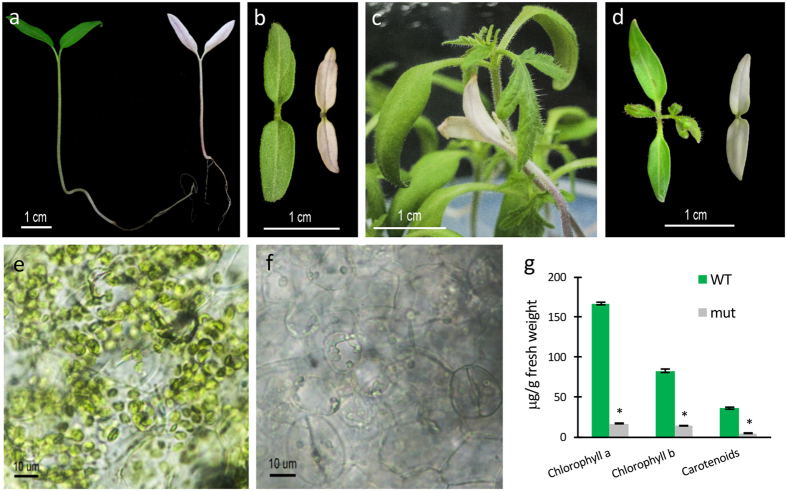
Phenotype of the *wls-2297* mutant. One-week-old wild type (left) and *wls-2297* seedlings (right) viewed from the side (**a**) and from above (**b**). Three-week-old wild type and mutant seedlings cultured *in vitro* on MS medium viewed from the side (**c**) and from above (**d**). Wild type (**e**) and mutant (**f**) cotyledons visualized using bright-field microscopy proving the lack of chloroplast in the mutant cells. (**g**) Chlorophyll and carotenoid content in wild type and *wls-2297* seedlings. Error bars are the standard error value from two biological replicates. Asterisk indicates significant differences between wild type control plants and mutant plants (Fisher’s Least Significant Difference test, *P* < 0.05).

A tomato T-DNA mutant collection generated using an enhancer-trapping construct was screened for developmental traits in TG2 plants grown in soil. As a result, the *wls-2297* mutant was selected due to the noteworthy albino phenotype of its cotyledons. After seed germination, mutant seedlings expanded cotyledons, but they did not fully elongate and never showed the characteristic green color associated with chlorophyll biosynthesis (Fig. 1a,b). Development of the shoot apical meristem was also arrested in the *wls-2297* mutant, as a few days after the cotyledons emerged seedlings experienced premature senescence and died without developing any true leaves. In general, albino phenotypes commonly result from alterations in the photosynthetic process, either directly affecting chloroplast development and functionality, or indirectly, as a consequence of mutations that promote significant defects in plant development or metabolism^31^. In a first step toward determining the metabolic pathway affected in the *wls-2297* mutant, seedlings were grown *in vitro* using a standard Murashige and Skoog (MS) culture medium supplemented with a high sugar concentration. Under these conditions, *wls-2297* mutant seedlings survived 3–5 days longer than in soil but finally died; during this period, they developed slower than wild type (WT) seedlings and developed no true leaves (Fig. 1c,d).

With a view to verifying whether the *wls-2297* mutation is involved in chloroplast development, fresh cotyledon tissues were observed under bright field microscopy, which revealed that cells of *wls-2297* cotyledons had an apparent lower density of chloroplasts than WT ones. This suggested decreased chlorophyll content of mutant cotyledons, in line with the pale green pigmentation of *wls-2297* chloroplasts as compared with WT ones (Fig. 1e,f). Therefore, chlorophyll and carotenoid contents were measured in WT and mutant cotyledons. As expected, results showed that levels of both chlorophylls and carotenoids were significantly lower in *wls-2297* mutant cotyledons (Fig. 1g), proving that the albino mutant phenotype was caused by a severe deficiency in photosynthetic activity.

### Molecular cloning and characterization of the *wls-2297* mutation

**Figure 2:**
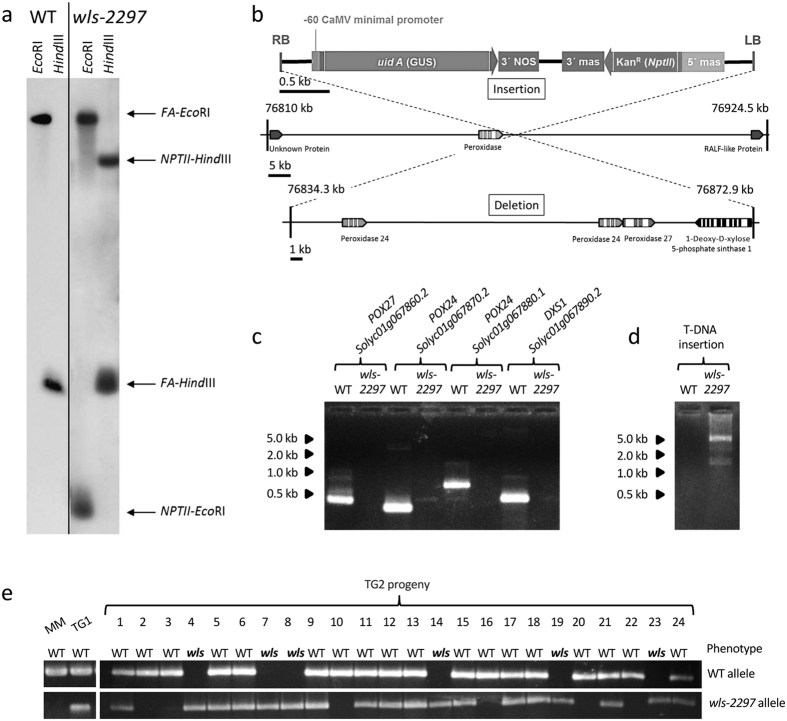
Molecular characterization of the *wls-2297* mutation. (**a**) Southern-blot hybridization of wild type (WT) and the *wls-2297* TG1 plant using the *FA-NPTII* probe. (**b**) Genomic re-organization caused by the T-DNA integration on the *wls-2297* genome. (**c**) PCR assays designed to detect the presence in wild type plant or the deletion in the *wls-2297* mutant of the three *POX* genes, the *DXS1* gene or (**d**) the T-DNA insertion in WT and the *wls-2297* mutant. (**e**) Co-segregation analysis performed in a TG2 family segregating for the *wls-2297* mutant phenotype. Cropped gels/blots are displayed in panels (**a**), (**c**), (**d**) and (**e**). Full-length gels/blots are included in Fig. S1.

Genetic analysis carried out on a segregating TG2 progeny of 121 plants indicated that the *wls-2297* mutant phenotype was inherited as a monogenic recessive trait (3:1 ratio, 84 WT:37 mutant; χ^2^ = 2.01; *P* = 0.16). This result agreed with that of Southern blot hybridization, which showed that a single T-DNA copy segregated in the *wls-2297* TG2 family here analyzed (Fig. 2a). Moreover, 88 TG2 seedling plants were screened *in vitro* for kanamycin resistance, and 22 of them displayed a *wls-2297* mutant phenotype. A complete co-segregation was found between *wls-2297* mutant and kanamycin resistance phenotypes in this segregating population (1:2:1 ratio; 22 mutant and kanamycin resistant:41 WT and kanamycin resistant:25 WT and kanamycin susceptible; χ^2^ = 0.61; *P* = 0.74), indicating that a single T-DNA insertion was responsible for the albino phenotype showed by the *wls-2297* mutant.

The genomic region flanking the T-DNA insertion was cloned and the sequences obtained were compared with the reference sequence of tomato genome (https://solgenomics.net). Results showed that the T-DNA is located on chromosome 1, the right border (RB) of the T-DNA being located at position 76834.3 kb, 1528 bp downstream of a gene encoding a putative PEROXIDASE enzyme (*POX, Solyc01g067850*). In addition, the left border (LB) of the T-DNA is placed at position 76872.9 kb, 217 bp upstream from the *DXS1* gene (*Solyc01g067890*) encoding for 1-deoxy-D-xylulose-5-phosphate synthase. According to this *in silico* analysis, the T-DNA insertion causes a 38.6 kb-deletion in the genome of the *wls-2297* mutant, which affects four genes, three putative genes encoding for POX enzymes (*POX24-Solyc01g067860, POX24-Solyc01g067870* and *POX27-Solyc01g067880*) and the *DXS1* gene (Fig. 2b).

In order to demonstrate that the 38.6 kb-deletion was the only mutation promoted by the T-DNA insertion and that no other chromosome rearrangements (e.g. translocation) occurred during mutagenesis, specific primers of the four genes located on the 38.6 kb genomic region were designed and used in PCR experiments to confirm their absence in *wls-2297* mutant plants. PCR products specific to the four reported genes from WT had the expected size and their sequences coincided with those reported in the tomato genome database (https://solgenomics.net), whereas no amplification products were obtained from the *wls-2297* mutant DNA (Fig. 2c). On the other hand, a long-PCR assay was performed to amplify the region between the two previously cloned T-DNA flanking regions. The long-PCR product obtained from *wls-2297* genomic DNA had the expected size of 5.2 kb (Fig. 2d) and its sequence confirmed the presence of a single T-DNA insertion causing a 38.6 kb-deletion in the genome of the *wls-2297* mutant. A co-segregation analysis performed using allele-specific primers designed from both the T-DNA and the genomic flanking sequences tagged in the *wls-2297* mutant revealed that the T-DNA insertion co-segregated with the *wls-2297* mutant phenotype (Fig. 2e). Taken together, these results provide strong evidence as to the genetic and molecular nature of the *wls-2297* mutant phenotype, which may be due to a 38.6 kb-deletion promoted by a single copy T-DNA insertion that affects three *POX* genes and the *DXS1* gene.

### Gene silencing experiments

**Figure 3:**
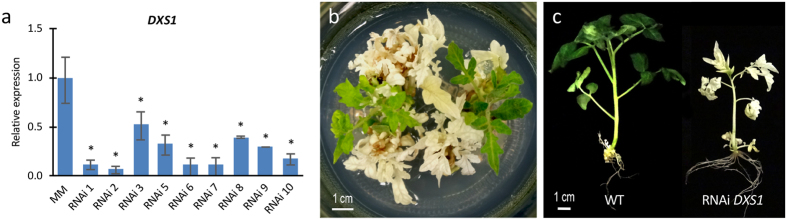
Silencing of *DXS1* causes an albino phenotype. (**a**) Expression analysis of the *DXS1* gene in the control (MM) and the RNAi *DXS1* lines. (**b**) Top view and (**c**) lateral view of albino RNAi transformants compared to wild type (WT) non-transformed plants. Error bars show the standard error value of two biological and two technical replicates. Asterisk indicates significant differences between control and RNAi *DXS1* lines (Fisher’s Least Significant Difference test, *P* < 0.05).

Given the role of the DXS1 enzyme in isoprenoid biosynthesis through the MEP pathway^18^ and the pigment measurements in the present work, the *DXS1* gene was considered the best candidate gene to be responsible for the *wls-2297* mutant phenotype. In order to demonstrate this hypothesis, we generated *DXS1* silencing lines in the cultivar Moneymaker using an RNA interference strategy (RNAi). After ploidy level determination, nine RNAi *DXS1* diploid lines showing a significant reduction of *DXS1* transcript levels (Fig. 3a) were further characterized under *in vitro* conditions. Interestingly, all *DXS1* repressed lines showed an albino phenotype, and even developed young leaves when sucrose was added to the MS medium, allowing explant development (Fig. 3b,c). Moreover, neither RNAi *DXS1* explants nor seedlings could be propagated since they died at early developmental stages. Therefore, the silencing of *DXS1* leads to similar developmental changes to *wls-2297* mutation, which provides strong evidence that the *wls-2297* mutant phenotype was due to the lack of *DXS1* gene function.

**Figure 4:**
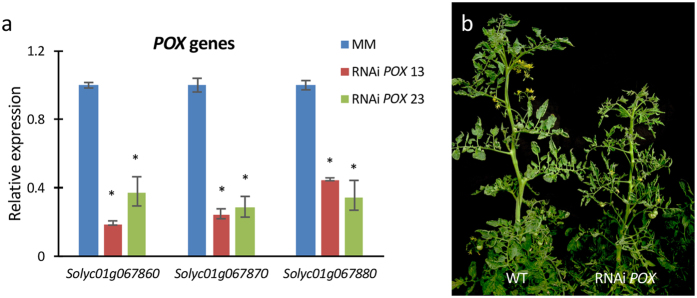
Silencing of *POX* genes does not generate an albino phenotype. (**a**) Expression analysis of the *POX* genes in the wild type control (MM, cv. Moneymaker) and two representative RNAi *POX* lines (RNAi *POX* 13 and RNAi *POX* 23). (**b**) Phenotype shown by wild type (WT) control non-transformed plants (left) and transgenic plant silencing the *POX* genes by RNAi (right). Error bars show the standard error value of two biological and two technical replicates. Asterisk indicates significant differences between control and RNAi *POX* lines (Fisher’s Least Significant Difference test, *P* < 0.05).

In addition, RNAi lines simultaneously silencing the three *POX* genes located in the deleted region were generated by using a gene construct that included a consensus sequence shared by the three *POX* messengers (Fig. S2b). Fifteen *POX* silencing diploid lines were selected for further analysis as they showed a significant decrease of *POX* transcripts ranging from 63 to 82% with respect to the control background (Fig. 4a). All of the RNAi *POX* lines developed normally, although plant vigor was slightly affected most likely due to peroxidase deficiency (Fig. 4b). Most importantly, none of these RNAi lines showed phenotype changes resembling those of the *wls-2297* mutant (Fig. 1a–d), which indicates that *POX* genes affected by the T-DNA insertion are not involved in the mutant phenotype here reported.

### Expression patterns of the *DXS* gene family

Tomato *DXS1* gene, which is deleted in the *wls-2297* mutant, is located on chromosome 1; according to the tomato genome database (https://solgenomics.net), it has a genome size of 4.2 kb and includes 10 exons and 9 introns. In addition to *DXS1*, two other tomato genes belonging to the *DXS* family have been identified, i.e. *DXS2*, located on chromosome 11 (*Solyc11g010850*) and *DXS3* located on chromosome 8 (*Solyc08g066950*), both also composed of 10 exons and 9 introns. The ORFs from the three genes, *DXS1, DXS2*, and *DXS3* code for proteins of 719, 714 and 709 amino acid residues, respectively (Fig. S3). Phylogenetic analysis performed with the three tomato DXS proteins as well as representative DXS protein of diverse plant species showed three independent groups of DXS proteins conserved among plants, i.e. DXS1, DXS2 and DXS3 clades (Fig. S4), a result which agrees with those previously described by Hofberger *et al*.^34^. In addition, the overall similarity among the three DXS tomato proteins ranges between 59 and 72%, DXS3 being the most divergent. The three proteins have conserved the three characteristic domains of the DXS family, these are an N-terminal thiamine pyrophosphate (TPP)-binding module, a pyrimidine binding domain and a transketolase C-terminal domain (Fig. S3).

**Figure 5:**
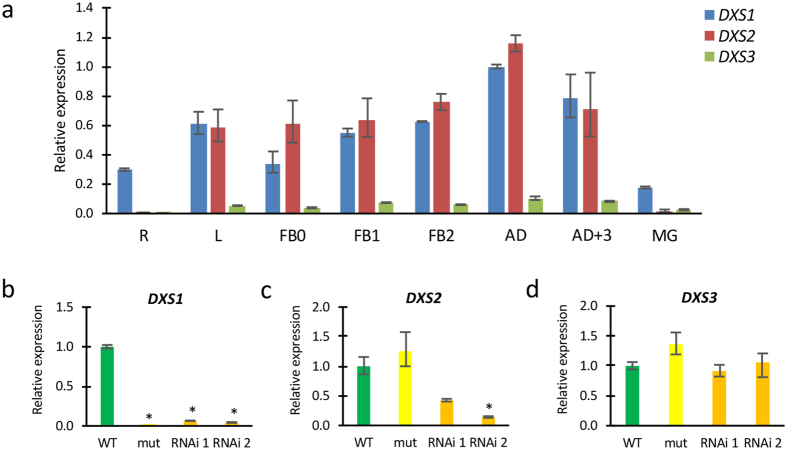
Expression analysis of the *DXS* genes. (**a**) The spatial expression of *DXS1, DXS2* and *DXS3* genes was analyzed by qRT-PCR in different organs of tomato plants. Expression analysis of the *DXS1* (**b**), *DXS2* (**c**) and *DXS3* (**d**) genes in wild type and *wls-2297* mutant seedlings, as well as two RNAi *DXS1* lines. R: root, L: leaf, FB0: Floral bud 0-3mm, FB1: Floral bud 4–7 mm, FB2: Floral bud 8–10 mm, AD: Anthesis day, AD + 3: Three days after anthesis, MG: Mature green fruit. Error bars show the standard error value of three biological and two technical replicates. Asterisk indicates significant differences between wild type control plants and mutant and silencing plants (Fisher’s Least Significant Difference test, *P* < 0.05).

With a view to analyzing the spatial expression pattern of the *DXS1* tomato gene, as well as the other two members of the *DXS* gene family, quantitative RT-PCR experiments have been carried out (Fig. 5a). A preliminary picture showed that all three *DXS* genes are expressed in a wide range of tissues, but a very low level of *DXS3* transcripts was found in comparison to *DXS1* and *DXS2* transcripts. *DXS1* expression was detected in both vegetative, i.e. root and leaf, and reproductive organs, including floral buds, flowers and fruits at several stages of development (Fig. 5a). *DXS1* transcript accumulation was high during flower development and later decreased sharply in fully developed green fruits (MG stage; Fig. 5a). Expression of *DXS2* was also found in leaf and flowers at several developmental stages, and particularly in flowers at anthesis; however, no *DXS2* transcripts were detected in roots, and only a very low number in MG fruits (Fig. 5a). These last two features, i.e. lack of expression in roots and a significant reduction of transcript level in MG fruits, also characterized the expression pattern shown by the *DXS3* gene (Fig. 5a).

The analysis of *DXS* transcript levels in the seedlings of the *wls-2297* mutant and in the *DXS1* RNAi silencing lines showed that *DXS1* expression was undetectable in the former, while it was silenced in the two RNAi lines analyzed (Fig. 5b). Though *DXS2* expression was not significantly affected in the *wls-2297* mutant, it decreased in the RNAi *DXS1* lines (Fig. 5c), despite the fact that the RNAi construct was specifically designed for silencing the *DXS1* gene (Fig. S2a). Finally, *DXS3* transcript levels were not significantly altered in the RNAi *DXS1* lines, but were slightly increased in *wls-2297* mutant seedlings (Fig. 5d).

### *In vivo* and molecular complementation of the *wls-2297* mutant phenotype

**Figure 6:**
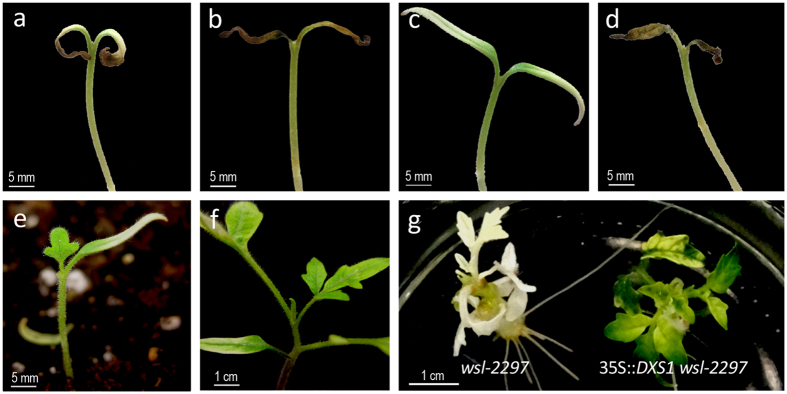
Complementation of the albino phenotype in the *wsl-2297* mutant. Two-week-old cotyledons of the *wsl-2297* mutant without any treatment (**a**) or with DXP present in the media (**b**) became necrotic and the plant died. (**c**) *wsl-2297* mutant seedlings treated daily with DXP on the shoot apical meristem became green but the plant died when the treatment with DXP was interrupted (**d**). (**e**,**f**) *wsl-2297* seedlings treated with DXP daily were able to develop small green leaves. (**g**) Explants from the *wsl-2297* mutant (left) and from *wsl-2297* mutant lines overexpressing *DXS1* (right).

To corroborate the functional role of *DXS1* gene in determining plant development and survival, two complementation experiments were performed. Thus, *in vivo* complementation of the *wls-2297* mutant phenotype was attempted by treatment of seedling plants grown under *in vitro* conditions with 1-Deoxy-D-xylulose-5-phosphate (DXP), the product of the DXS enzyme activity. When DXP was added to the *in vitro* culture media, mutant seedlings not only did not recover the wild type phenotype, rather they died even faster than *wls-2297* mutant ones (Fig. 6a,b). On the contrary, daily treatment with DXP on the shoot apical meristem led the mutant seedlings to develop green live tissues from those cells which incorporated DXP (Fig. 6c), and they were even able to develop small green leaves (Fig. 6e,f), suggesting that DXP is essential for photosynthetic activity of plant tissues. Nevertheless, it is noteworthy that plant development ceased and seedlings died a few days after DXP treatment ceased (Fig. 6d).

In addition, the *DXS1* gene was constitutively expressed under a 35S promoter in *wls-2297* mutant seedlings to ascertain whether increased *DXS1* transcript levels were able to rescue the wild type phenotype. Results showed that transgenic explants develop green tissues from albino tissue sections (Fig. 6g), confirming that the lack of photosynthetic activity displayed by the *wls-2297* albino mutant can be firstly reverted by *DXS1* expression. However, as the overexpressing *DXS1* explants grew, tissues turned yellow and finally acquired an albino phenotype and died.

### Effects of *DXS1* overexpression on tomato fruit pigmentation

**Figure 7:**
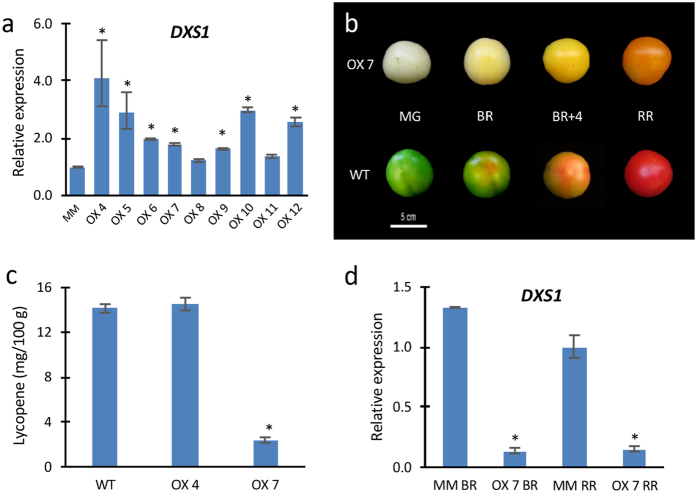
Overexpression *DXS1* lines. (**a**) *DXS1* transcript levels in young leaves from 35S::*DXS1* lines with respect to wild type untransformed cv. Moneymaker plants. (**b**) Fruit phenotypes at four developmental stages, i.e. mature green (MG), breaker (BR), 4 days after breaker (BR + 4) and red ripe (RR) of 35S::*DXS1* line OX7 (above) and wild type (WT) (below). (**c**) Lycopene content of fruits from WT and 35S::*DXS1* lines OX4 and OX7. (**d**) Relative expression of *DXS1* in fruits (BR: breaker; RR: Red Ripe) of OX7 line and wild type. Error bars show the standard error value of two biological and two technical replicates. Asterisk indicates significant differences between control and 35S::*DXS1* lines (Fisher’s Least Significant Difference test, *P* < 0.05).

It has been proposed that the protein coded by *DXS1*, the plastidic form of 1-Deoxy-d-xylulose-5-phosphate synthase, is the limiting enzyme for isoprenoid biosynthesis^26^. In order to determine whether the *DXS1* gene is a limiting step in the MEP pathway of tomato plants, nine diploid transgenic lines overexpressing *DXS1* cDNA (OX lines) were generated in the cultivar Moneymaker using a constitutive 35S promoter gene construct, and subsequently characterized in greenhouse conditions (Fig. 7). With the exception of the OX7 line, no changes in the colour and lycopene concentration were observed in the fruits of 35S::*DXS1* lines even though they expressed the *DXS1* gene at a higher level than control plants (Fig. 7a). Contrary to expectations, tomato fruits yielded by the OX7 line showed a pale colour during the whole ripening process (Fig. 7b). The lack of red colour in mature fruits was most probably due to the low level of lycopene pigment detected in the OX7 transgenic line (Fig. 7c). Analysis of *DXS1* relative expression carried out in tomato fruits at breaker (BR) and red ripe (RR) stages of development showed that *DXS1* was not overexpressed, but rather it was repressed in these OX7 fruits, suggesting that the pale colour and low lycopene concentration displayed by this line was caused by the post-transcriptional gene silencing (PTGS) of the *DXS1* gene (Fig. 7d). Indeed, PTGS triggered by the 35S promoter have widely been reported in transgenic plants^35^. However, the fact that T-DNA integration site in the OX7 line might be responsible for the altered phenotype cannot be definitively ruled out.

### Influence of *DXS1* overexpression and silencing on the MEP pathway

The MEP pathway operates through the participation of eight consecutive enzymes to generate IPP and DMAPP, which serve as the basis for the biosynthesis of all isoprenoid compounds^36^,^37^. To investigate the effect of *DXS1* overexpression and silencing on the MEP pathway, the expression of the genes participating in this pathway was analyzed by qRT-PCR in the three transgenic lines showing either the highest or lowest *DXS1* expression levels (Fig. S5a).

Within the MEP pathway, DXS generates DXP by the transketolase-type condensation of pyruvate and D-glyceraldehyde 3-phosphate, which is converted to MEP by the enzyme coded by the *DXP* reductoisomerase gene (DXR, *Solyc03g114340*), whose expression profile remained quite similar in wild type and *DXS1* overexpression lines (Fig S4b). However, *DXS1* RNAi lines showed a decreased tendency in *DXR* transcript level compared to wild type plants (Fig S4b). Subsequently, MEP is converted into IPP and DMAPP by the consecutive action of five independent enzymes: i.e. 2-C-methyl-D-erythritol 4-phosphate cytidyltransferase (MCT, *Solyc01g102820*), 4-diphosphocytidyl-2-C-methyl-D-erythritol kinase (CMK, *Solyc01g009010*), 2-C-methyl-D-erythritol 2,4-cyclodiphosphate synthase (MDS, *Solyc08g081570*), 4-hydroxy-3-methylbut-2-en-1-yl diphosphate synthase (HDS, *Solyc11g069380*), and 4-hydroxy-3-methylbut-2-enyl diphosphate reductase (HDR, *Solyc01g109300*). Expression analysis carried out in *DXS1* overexpression lines showed similar expression profiles between wild type and transgenic lines in the transcript levels of the genes coding for these five enzymes (Fig. S5c–g). On the other hand, the transcript levels of *MCT, CMK* and *MDS* genes were remarkably reduced in *DXS1* RNAi lines (Fig. S5c–g). However, *HDS* and *HDR* genes showed certain variability in expression levels across these silencing lines Fig. S5c–g).

In the final step of the MEP pathway, geranylgeranyl diphosphate (GGPP), a common precursor for the synthesis of phyllochinones, tocopherols, plastoquinones, chlorophylls, gibberellins and carotenoids, is generated by geranylgeranyl diphosphate synthase (GGPS, *Solyc11g011240*) that catalyzes the condensation of three IPP and one DMAPP units. Expression levels of the *GGPS* gene in *DXS1* RNAi lines showed variable levels of expression (Fig. S5h), similar to those observed for *HDS* and *HDR* genes; whereas slight differences were found in *DXS1* overexpression lines compared to wild type plants (Fig. S5h).

## Discussion

In recent decades, insertional mutant collections have been particularly useful for identifying and characterizing genes of interest in different model species such as Arabidopsis, rice and tomato^38^,^39^,^40^. This work reports the isolation and functional analysis of the tomato *wls-2297* T-DNA mutant, whose characterization led us to demonstrate once again the value of T-DNA insertional mutant libraries for gene discovery in a species of agronomic interest such as tomato. The *wls-2297* mutant was selected based on its albino phenotype and the arrested development at expanded cotyledon stage. Molecular analyses performed on the *wls-2297* mutant revealed that the observed albino phenotype is due to a single T-DNA insertion, whose integration in the tomato genome caused a 38.6 kb-deletion at chromosome 1 that resulted in the loss of *DXS1* and three *POX* genes. Phenotypic and expression analyses of RNAi silencing lines led us to conclude that *wls-2297* is a knock-out mutant affected in the *DXS1* gene, and that the loss of DXS1 activity is responsible for the *wls-2297* mutant phenotype. Indeed, this result was further confirmed by means of both *in vivo* complementation assays with DXP and *DXS1* overexpression on the *wls-2297* mutant, which partially rescued the albino phenotype. At early stages of plant development, the *wls-2297* phenotype was completely rescued; however, over time the leaves of the *wls-2297* mutant plants overexpressing the *DXS1* gene turned yellow, became albino and eventually died prematurely. Functional assays based on *DXS1* overexpression showed dissimilar results in different plant species, ranging from no changes in isoprenoid content^28^,^41^ to unequal increases in various isoprenoid compounds including total chlorophylls and carotenoids^26^,^29^. Overall, these previous works have proved that constitutive *DXS1* overexpression does not necessarily mean high levels of isoprenoid end products. Indeed, it has been found that *DXS1* transcript levels in overexpression lines, and therefore isoprenoid levels, are usually not so high as might be expected^26^,^28^,^29^,^41^. In this study, expression analysis of the MEP pathway genes suggested that *DXS1* overexpression did not significantly alter transcript profiles of the MEP pathway since gene expression levels in *DXS1* overexpressing lines were quite similar to those found in wild type plants (Fig. S5). However, different expression profiles of the MEP pathway genes were found in the *DXS1* silencing lines. Thus, the expression levels of *DXR, MCT, CMK* and *MDS* genes were significantly reduced in albino RNAi transformants compared to wild type non-transformed plants (Fig. S5b–e), suggesting a role for DXS in transcriptional regulation of the first steps of the MEP pathway. On the other hand, expression profiles of *HDS, HDR* and *GGPS* genes showed an evident variation among different *DXS1* RNAi lines (Fig. S5f–h). Most likely, the explanation for these results relies on the complex regulation of the MEP pathway, which occurs at both transcriptional and post-transcriptional levels^42^ to ensure its proper functioning. Due to this pathway complexity, it has been suggested that elevated levels of *DXS1* transcripts can only exert an influence on the MEP pathway when adequate precursors from intermediary metabolism are accessible^28^. Given that levels of the different isoprenoids do not change equally in *DXS1* overexpressing lines^26^,^28^,^29^,^41^, the fact that overexpression of *DXS1* did not completely rescue the albino phenotype of the *wls-2297* mutant might be due to disproportionately altered isoprenoid levels. However, the possibility that some *wls-2297 DXS1* overexpressing lines here reported may undergo co-suppression effects of *DXS1* expression cannot be discarded, as it is a commonly reported phenomenon in genetic transformation experiments using strong constitutive promoters^35^. Unfortunately, we were not able to perform expression studies to corroborate this hypothesis, as 35S::*DXS1* plants died prematurely.

In Arabidopsis, the *DXS1* knock-out mutants *cla1-1*^31^ and temperature-sensitive allele *chs5*^30^ also showed an albino phenotype as well as altered plant development. When both mutants were grown in a nutrient medium supplemented with glucose, they were able to sustain their development while maintaining their albino phenotype. Thus, *chs5* plants developed slowly but reached a similar size to wild type plants^30^, whereas the *cla1-1* mutant even generated floral buds, which did not however manage to produce normal flowers^31^. In this study, tomato RNAi *DXS1* lines developed three-four albino leaves, probably as a consequence of residual *DXS1* expression; however, the *wls-2297* mutant did not develop true leaves, but rather its growth was arrested at the fully expanded cotyledon stage or slightly later when grown in a nutrient medium supplemented with a high sugar concentration. These results suggest a significant difference between DXS1 action mechanisms in Arabidopsis and tomato. Araki *et al*.^30^ suggested that a plant with a loss of DXS activity did not survive past the seedling stage due to the lack of thiamine, one of the first branches of the MEP pathway^43^. They therefore proposed that the partial development of albino *chs5* plants was facilitated by other *DXS* genes^30^. Arabidopsis *DXS1* and *DXS2* genes show high homology and are both included in the subfamily DXS1^34^, suggesting that they would have redundant functions. However, the Arabidopsis *DXS2* gene has recently been renamed as *DXL1*, which might not have DXS activity, but rather has acquired a novel, as yet unknown biochemical function^32^. In contrast, the Arabidopsis *DXS3* gene has been grouped within a clade composed of highly divergent protein sequences^20^, which lack many of the highly conserved residues known to be essential for DXS activity^44^. Nonetheless, marginal, though significant, DXS activity has recently been reported for the corresponding ortholog DXS3 in maize^20^. Hence, if the DXS3 function was conserved in other species, the survival of Arabidopsis *chs5* and *cla1-1* mutants might be explained by the DXS activity of the DXS3 enzyme. As in Arabidopsis, the tomato DXS family also consists of three genes, but in contrast to Arabidopsis, tomato *DXS* genes code for proteins belonging to each one of the proposed DXS clades (Fig. S4), and in addition they have different expression patterns. The tomato *DXS1* gene is ubiquitously expressed and its highest transcript levels are observed during fruit ripening^34^,^45^. However, *DXS2* transcripts are abundant in only few tissues, including young leaves and flowers, as well as isolated trichomes^34^,^45^. On the other hand, *DXS3* transcript levels are extremely low compared to the other two tomato *DXS* genes according to previous results^34^. The roles of tomato DXS1 and DXS2 have been described without overlapping function in modulating isoprenoid metabolism^16^,^22^,^33^,^45^. Our results are in agreement with this hypothesis, since DXS2 and DXS3 activities did not compensate for the loss of DXS1 function in the *wls-2297* mutant, indicating that DXS1 plays a vital role in the development of tomato plants.

Since the non-mevalonate pathway of isoprenoid biosynthesis was described, the exchange of isoprenoid intermediate compounds between MVA and MEP pathways has been a matter of some dispute. Certain evidence of cross-talk between MVA pathway in the cytosol and MEP pathway in the plastid has been reported in different plant species, including Arabidopsis and a solanaceous species like tobacco^46^,^47^,^48^. However, it has also been proposed that a tightly regulated cellular compartmentalization of MVA and MEP pathways occurs during isoprenoid biosynthesis^28^. In addition, a tissue-dependent regulation of tomato MEP genes has recently been reported supporting the complexity of this pathway^49^. Characterization of the *wls-2297* mutant, which displays a complete inhibition of DXS activity due to a loss-of-function of the *DXS* gene, showed a drastic reduction in chlorophyll and carotenoid biosynthesis (Fig. 1g), a feature that should not be detected if there were an active exchange of isoprenoid intermediates between both pathways. Therefore, our results not only support that the MEP pathway is essential for photosynthetic pigment biosynthesis, but also that functional cross-talk with the MVA pathway may not occur at least during the first steps of isoprenoid biosynthesis. Although periodic supplementation of *wsl-2297* mutant seedlings with DXP during several days promoted minor development, it did not avoid death of the seedlings once the treatment ceased. This result suggests that if an exchange of isoprenoid intermediates such as IPP and DMAPP occurs in tomato chloroplasts, it must be at a rate that is insufficient to allow plant growth. In addition, this hypothesis agrees with the blocking of plastidial isoprenoid biosynthesis observed after treatment with fosmidomycin, a specific inhibitor of DXP reductoisomerase, the enzyme catalyzing the previous step to that of DXS in the isoprenoid biosynthetic pathway^50^. Taken as a whole, these results regarding the lethality of the *wls-2297* mutation, also found in *DXS1* silencing lines, prove that the *DXS1* gene is a crucial genetic factor for plant organ growth and survival.

## Methods

### Plant material and growth conditions

The *wls-2297* mutant was isolated from a T-DNA insertional collection, which was generated from the tomato (*Solanum lycopersicum* L.) cultivar Moneymaker through infection with *Agrobacterium* strain LBA4404 containing the enhancer trap vector pD991^51^ (kindly supplied by Dr. Thomas Jack; Department of Biological Sciences, Dartmouth College, USA). The TG1 line was self-pollinated to obtain the TG2 progeny, which was grown in soil conditions (a mixture of peat and coconut fiber (1:1) and, after sowing, a cover of vermiculite). Due to the fact that the *wls-2297* mutant homozygous plants did not survive enough to achieve fruit set, *wls-2297* line were perpetuated by self-pollination of wild type heterozygous plants growth in soil under greenhouse conditions using standard practices with regular addition of fertilizers. Seedlings analysed in this project were grown under standard conditions (16 h light/8 h darkness, ≈ 25 °C, 60% RH) in a growth chamber. Co-segregation studies of the mutant phenotype with kanamycin resistance conferred by the *NEOMYCIN PHOSPHOTRANSFERASE III (NPTII*) marker gene was carried out by sowing seeds on Murashige and Skoog^52^ (MS) agar medium supplemented with sucrose (10 g l^−1^) and kanamycin (100 mg l^−1^). The sodium salt of 1-Deoxy-D-xylulose-5-phosphate (DXP, Sigma-Aldrich) resuspended in water (1 mg/mL) was used to perform *in vivo* complementation of the albino phenotype in the *wls-2297* mutant.

### Microscopy analysis of chloroplasts and pigment measurement

For observation of chloroplasts/plastids in living leaf tissues, a section of tomato cotyledon was excised and its cuticle removed manually. Then, these cotyledons were examined using a bright field microscope Nikon Optiphot-2 equipped with Nikon Digital Camera. The chlorophylls and carotenoids concentrations were determined by UV-VIS Spectroscopy following the method described by Lichtenthaler and Buschmann^53^.

### DNA isolation and DNA-blot hybridization

Genomic DNA was isolated from 100 mg of young leaves using Plant DNAzol Reagent (Invitrogen) following the instructions provided by the manufacturer. In order to determine the number of T-DNA insertions introduced in the *wls*-*2297* mutant, a DNA-blot hybridization was carried out from 15 μg of genomic DNA digested by restriction enzymes *Eco*RI and *Hin*dIII, electrophoresed through-out a 0.8% agarose-TBE gel, blotted onto Hybond N+ membrane (Amersham) and hybridized with a *FA*-*NPTII* radioactive probe, which was labelled with ^32^P by random priming with the Rediprime II Random Prime Labelling System (Amersham). Nylon membranes hybridized were exposed to Hyperfilms (Amersham). The *FA-NPTII* probe was synthetized including the complete coding sequence of the tomato *FALSIFLORA (FA*) gene, which was employed as hybridization positive control, and the *NPTII* gene used as a selective marker in the T-DNA.

### Cloning of T-DNA flanking sequences and PCR genotyping

Sequences flanking the *wls-2997* T-DNA insertion were cloned by amplification with a single-side specificity (anchor-PCR) assay described by Loh^54^. With this end, genomic DNA was digested with restriction endonucleases that generate blunt ends, i.e. *Alu*I, *Dra*I, *Eco*RV, *Hin*cII, *Pvu*II, *Sca*I, *Stu*I and *Sma*I (Takara Bio company). After digestion, adaptors were linked to the digested genomic DNA restriction fragments. The fragments linked to adaptors were then amplified by three assembled PCR with specific primers for the Right Border sequence (Anchor Right-1, Anchor Right-2 and Anchor Right-3; Table S1) or for the Left Border (Anchor Left-1, Anchor Left-1 and Anchor Left-1; Table S1) from the T-DNA, combined with adaptor specific primers Adaptor-1, Adaptor-2 and Adaptor-3 (Table S1). PCR amplification products were purified using GenElute PCR Clean-up Kit (SIGMA Aldrich) and sequenced in an Applied Biosystems 3500 Genetic Analyzer. The cloned sequences were compared with SGN Database (https://solgenomics.net) to assign the T-DNA insertion site on tomato genome. Co-segregation of the T-DNA insertion site with the *wls-2997* phenotype was evaluated by PCR using i) the specific genomic forward and reverse primers to amplify the wild type allele (without T-DNA insertion) and ii) one specific genomic primer and the specific T-DNA primer to amplify the mutant allele (carrying the T-DNA insertion). The sequence of genotyping primers used is listed in Table S1.

Specific primers for *DXS1 (Solyc01g067890*) and the three *POX (Solyc01g067860, Solyc01g067870, Solyc01g067880*) genes were designed (Table S1) and used in PCR experiment to demonstrate that a 38.6 kb-deletion is on *wls-2297* mutant plants. Amplification was performed using in a volume of 30 μl using 10 ng of total DNA, 50 ng of each primer, 0.25 mM dNTPs, 2.5 mM MgCl_2_, and 1 U BIOTAQ^TM^ DNA Polymerase (Bioline) in 1X Taq buffer. DNA was amplified under the following thermal cycling conditions: 94 °C for 5 min, followed by 35 cycles at 94 °C for 30 s, 60 °C for 30 s, and 72 °C for 2 min, and a final extension of 5 min at 72 °C. PCR products were analysed in 1% agarose gels in 1X SB buffer (200 mM NaOH, 750 mM boric acid, pH 8.3) and visualized with ethidium bromide. In addition, to amplify the genomic region located between the T-DNA flanking regions a Long-PCR assay was carried out using Elongase^®^ Enzyme Mix (Invitrogen) following the instructions given by the manufacturer. The primers used in this experiment were Genotyping-F1 and Genotpyping-R2 (Table S1). Genomic DNA from wild type (cv. Moneymaker) and the *wls-2297* mutant plants was used as template.

### RNA preparations and gene expression analysis

Total RNA was purified from using Trizol reagent (Invitrogen) following the manufactures instructions. Contamination of genomic DNA was removed by using the DNA-free^TM^ kit (Ambion), and cDNA was synthetized from the DNA-free total RNA by means of a M-MuLV reverse transcriptase (Invitrogen) using as primers a mixture of random hexamer and 18-mer oligo(dT).

Expression analysis were performed by real-time PCR assays using the SYBR Green PCR Master Mix kit in a 7300 Real-Time PCR System (Applied Biosystems, Foster City, CA, USA) and a pair of specific primer pairs for each gene analysed (Table S1). Data obtained were collected and analysed using the System Sequence Detection Software v1.2. Results were calculated using ΔΔCt method by comparison to a data point from the wild type samples and are expressed in arbitrary units. The housekeeping *UBIQUITINE3* gene was used as an internal reference in the gene expression analyses performed. Absence of genomic DNA contaminating in the RNA sample analysed by RT-PCR was demonstrated as previously described^55^. Mean comparison (Fisher’s Least Significant Difference test, LSD) was used to determine significant differences among gene expression levels. Analyses were performed using the Statgraphics Centurion XVI software package and data presented as means ± standard error.

### Generation of DXS transgenic tomato plants

The *DXS1 (Solyc01g067890*) complete open reading frame (ORF) was amplified from tomato cv. Moneymaker cDNA using a pair of specific primers 35SDXS1F and 35SDXSR to introduce a *Bam*HI restriction site 127 bp upstream the initiation codon and a *Kpn*I restriction sites downstream the stop codon (Table S1). The PCR product was cloned into a p-GEM^®^-T-Easy plasmid and sequenced in order to confirm the absence of mutations in the amplicon. The selected plasmid was digested with *Bam*HI and *Kpn*I, and the *DXS1* cDNA was subcloned under the control of the constitutive CaMV promoter together a plant kanamycin resistant selection marker into the binary vector pROKII to generate an overexpression gene construct used to transform plants of the cv. Moneymaker. The same *DXS1* cDNA was subcloned under the control of the constitutive CaMV promoter together with a plant hygromycin resistant selection marker into the binary vector pCAMBIA1302 to generate an overexpression gene construct used to complement the *wls-2297* mutant seedlings.

In order to silencing the *DXS1* gene, an interference RNA (RNAi) approach was followed. A 330 bp specific fragment of the *DXS1* cDNA was amplified, using primers RNAiDXS1F, to introduce a *Xba*I and a *Xho*I restriction sites, and RNAiDXS1R, to introduce a *Cla*I and a *Kpn*I restriction sites (Fig. S2a; Table S1), and cloned into p-GEM^®^-T-Easy. The insert of RNAiDXS1 was liberated from the p-GEM^®^-T-Easy twice, firstly by *Xho*I and *Kpn*I digestion and secondly by *Xba*I and *Cla*I digestion. Both inserts liberated were cloned as inverted repeats into vector pKannibal. The resulting plasmid was digested with *Not*I and fragment isolated was cloned into the binary vector pART27 to express inverted repeat sequences of *DXS1* separated by intronic sequences under the control of the constitutive promoter 35S. In order to generate RNAi lines to inhibit the three *POX* genes (*Solyc01g067860, Solyc01g067870, Solyc01g067880*) simultaneously, a pair of primers annealing in a conserved domain for the three POX, RNAiPOXsF and RNAiPOXsR (Fig. S2b; Table S1), were used to generate the silencing construct obtained as described for the *DXS1* gene.

Overexpression and silencing binary vectors were electroporated into *Agrobacterium tumefaciens* and Agrobacterium-mediated transformation of Moneymaker tomato cultivar cotyledons was performed following the protocols previously described^56^. The ploidy level in transgenic plants was evaluated by flow cytometry according to the protocol described by Atarés *et al*.^57^ and diploid transgenic lines were selected for further phenotypic and expression analyses.

## Additional Information

**How to cite this article:** García-Alcázar, M. *et al*. Albino T-DNA tomato mutant reveals a key function of 1-deoxy-D-xylulose-5-phosphate synthase (DXS1) in plant development and survival. *Sci. Rep.* **7**, 45333; doi: 10.1038/srep45333 (2017).

**Publisher's note:** Springer Nature remains neutral with regard to jurisdictional claims in published maps and institutional affiliations.

## Supplementary Material

Supplementary Material
