# Supplementary material for: Albino T-DNA tomato mutant reveals a key function of 1-deoxy-D-xylulose-5-phosphate synthase (DXS1) in plant development and survival
Source: Sci Rep. 2017 Mar 28;7:45333. doi: 10.1038/srep45333 (PMC5368609; doi:10.1038/srep45333)
Supplement: Supplementary Material [file srep45333-s1.pdf]

Albino T-DNA tomato mutant reveals a key function of 1-deoxy-D-xylulose-5-phosphate synthase (DXS1) in plant development and survival

Manuel García-Alcázar[1,#], Estela Giménez[1,#], Benito Pineda[2], Carmen Capel[1], Begoña García-Sogo[2], Sibilla Sánchez[2], Fernando J. Yuste-Lisbona[1], Trinidad Angosto[1], Juan Capel[1], Vicente Moreno[2] and Rafael Lozano[1,*]

[1] Centro de Investigación en Biotecnología Agroalimentaria (BITAL). Universidad de Almería, 04120 Almería, Spain.

[2] Instituto de Biología Molecular y Celular de Plantas (UPV-CSIC), Universidad Politécnica de Valencia. 46022 Valencia, Spain.

[#] These authors contributed equally to this work.

[*] Corresponding author: rlozano@ual.es

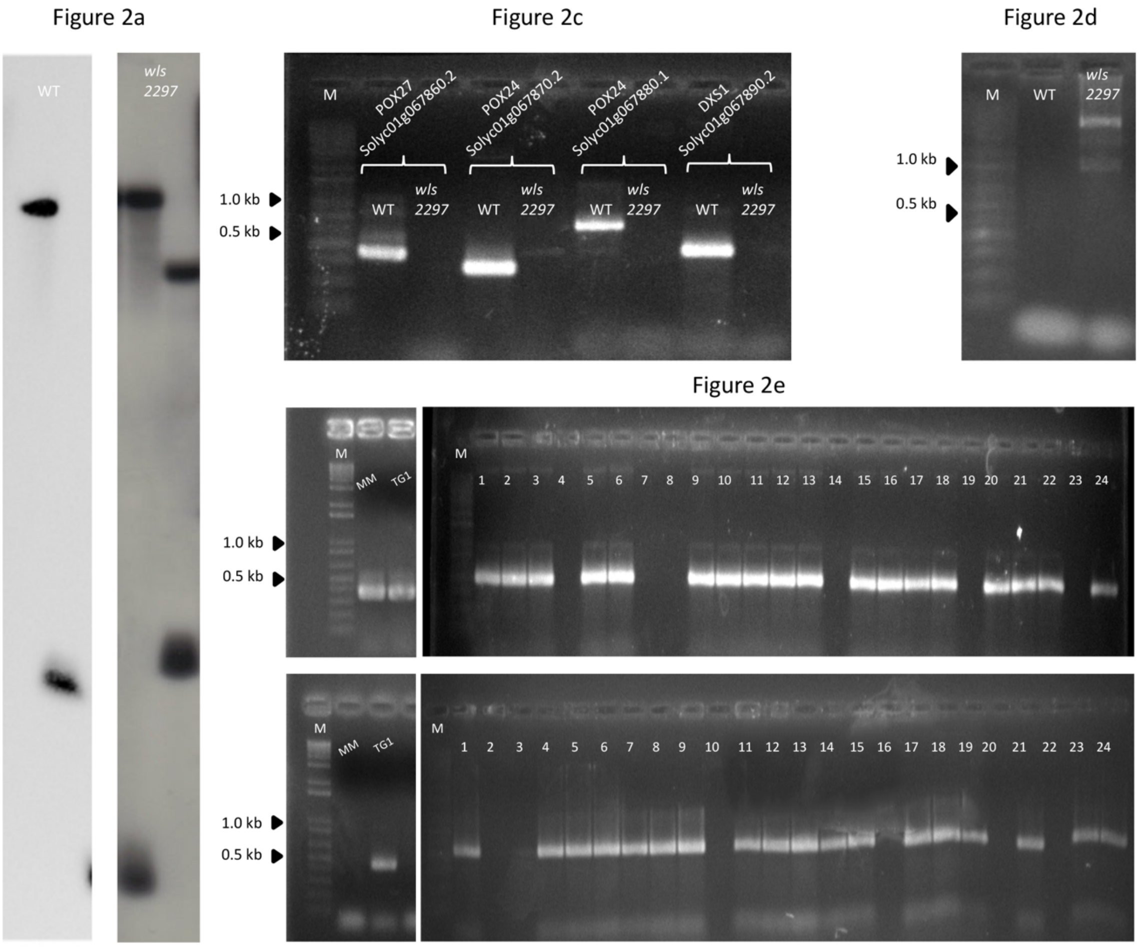

Figure S1. Full-length gels/blots of cropped images shown in Figure 2.

a

```
DXS1        CCTTCACATTCCTCCACATTGGATCAACACCATTTGATCCTT-------TT-----TATC
DXS2        TCCTTGTAGTGTTAAAAAATAAGTAAATTAAATT--ACCATG-------TTGAGAATATA
DXS3        ATTTTAAACAATGAAACAAT-AATAAATATAATAGAAATATTGGTGTAATTAGTTATCTA
               *   *       * * *   * **    **   *   *        **     * *

DXS1        AAAGATTCATTTT-------------------TTTT----TTCACTTACCCTCCAAT---
DXS2        GACAATTTATCAT-------------------TTTGGGAATTGATATTTTTTCTATT---
DXS3        GACAAGCCCTTCTCCGACAAACCAAAGTGATATTTG---ACCCATATACTTTTCATTCAC
             *  *    *  *                   ***        *  *    *  * *

DXS1        ------TACATCTTCATAAA-----CAACATTTTAGTGACAGTAGCACCAA--CACACCC
DXS2        ------TTTATATTTAAGGAAGTTGCTACTCTTTCTTGTTTGTAACTATAAATTATGTCC
DXS3        CATCACTACCTCTCCAGAAA------TGCTCTGTTTGTCCTGT----------TATACGC
                  *   * *  *   *        *  * *       **           *    *

DXS1        CACTAGAATTTTCTTGAAGTAAACCCCTTTTTTCAAGAATCAAGAAACCAC-TTATAAAA
DXS2        CCCT----TTTTCTC--------TTCTTGTGTCTAAAAATAATTGAACAAC-TTGTTCAT
DXS3        CATT---------TTCGAGTTGATTTTTTTTCTGAAAGCTCTCAATCTCATACTCCAAAC
            *  *         *             * *    **   *         *   *

DXS1        TTTGTGGGTTTTCATTGAAACAAAGGAAAAAAAACAGTTGAATTGACTAATCATGGCTTT
DXS2        TTTCT---CTCTGACTAAAATTTTTCATACAAAA------------------ATGGCAGT
DXS3        TTCGT---TTTTTACCGAATTGAGTGG-------------------------ATGGTTGC
            **  *    * *  * **                                  ****

DXS1        GTGTGCTTATGCATTTCCTGGGATTTTGAA-----CAGGACTGGTGTGGTTTCAGATTCT
DXS2        TTCTTCAGGCACTGT-------ATTTAGAATCAACCAGCAC----------CCAAGTTCT
DXS3        -TGTTACTGCTCAGTACCC---ATTTGGTATCTGCC---------------CTCAGTTC-
             * *       *  *       **** * *     *                    ***
DXS1        TC---TAAGGCAACCCCTTTGTTCTCTGGA
DXS2        CCATACATGAAAACTCCAAGATTTT----A
DXS3        -----CATGGGAATTCAAGGTTACT-----
                    *  **  *     *  *
```

b

```
Solyc01g067860      CATTTGATAGCAATTATTTCAACATTCTTATTAATCAAAACAAAGGGTTGTTCCAATCAG
Solyc01g067870      CATTTGATAGCAATTATTTCAACATTCTTATTAATCAAAACAAAGGGTTGTTCCAATCTG
Solyc01g067880      CATTTGATAGCAATTATTTCAACATTCTTATTAATCAAAACAAAGGGTTGTTCCAATCTG
                    ********************************************************** *

Solyc01g067860      ATGCTGCACTTCTCAATGACAAAGACTCAGTAATTGTCATCAAGAAATTACTAAAGACA
Solyc01g067870      ATGCTGCACTTCTCAATGACAAAGACTCAGTAATTGTCATCAAGAAATTGCAAGATGATA
Solyc01g067880      ATGCTGCCTTTCTCAATGACAAAGACTCAGTAATTGTCATCAAGAAATTGCAAGATGATA
                    *******  **************************************** * * * ** *

Solyc01g067860      AGACTTTCTTTATTGAGTTTGCAAAATCCATGAAGAAAATGGAGCCATT
Solyc01g067870      ACACTTTCTTTTCCGAGTTTGCGAAATCCATGAAGAAAATGGAGCCATT
Solyc01g067880      ACACTTTCTTTTTCGAGTTTGCGAAATCCATGAAGAAAATGGAGCCATT
                    * *********   ******** ***************************
```

Figure S2. Sequences used for the RNAi constructs. (a) In order to silence only the *DXS1* gene, specific primers for this gene (highlighted in yellow) were designed to amplify a fragment of the coding sequence of *DXS1* with low homology to the sequences of *DXS2* and *DXS3* genes. (b) In the case of the three peroxidase genes, primers (highlighted in yellow) were designed to amplify a 161 bp length genomic region of *Solyc01g67860* with high homology (95.5%) to *Solyc01g67870* and *Solyc01g6780*, the two other peroxidase coding genes located in the genomic fragment deleted in the *wls-2297* mutant.

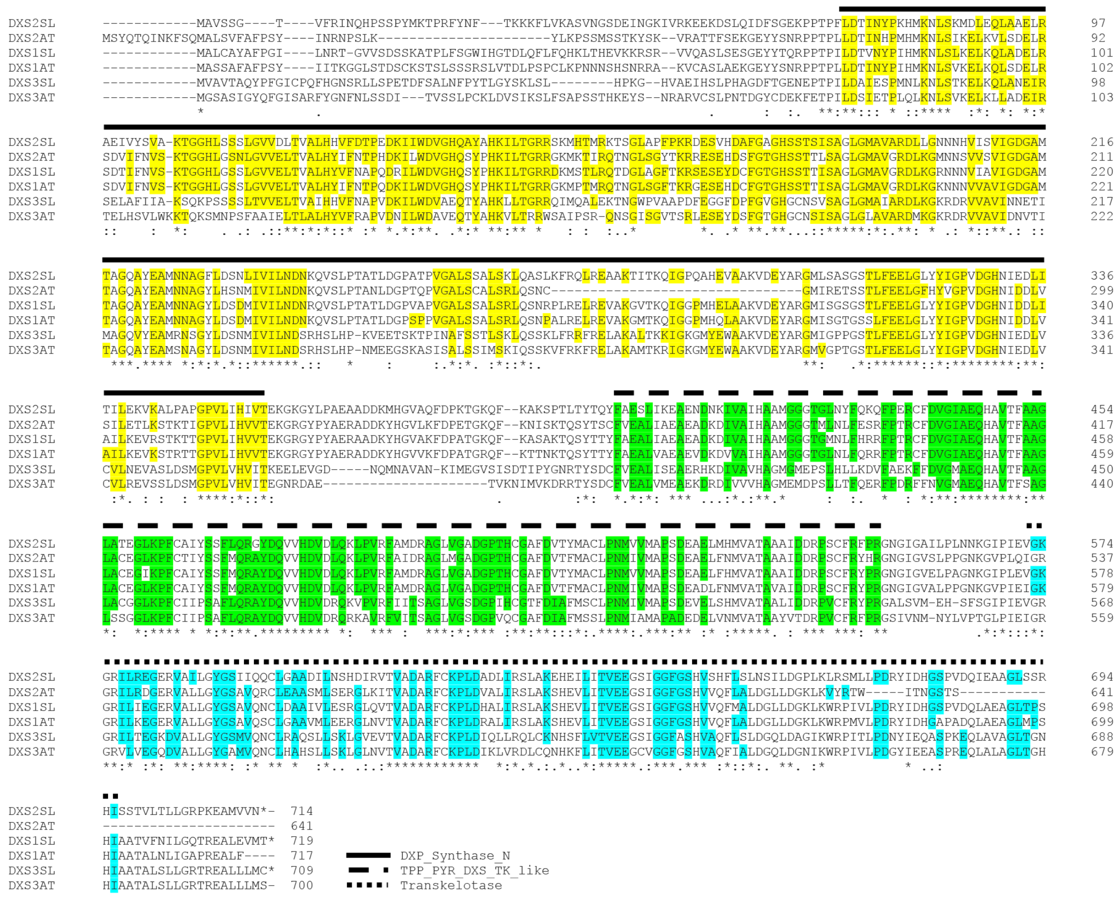

Figure S3. Multiple alignment of DXS proteins of tomato and Arabidopsis. Alignment of tomato and Arabidopsis DXS1, DXS2 and DXS3 proteins performed with ClustalW with conserved domains highlighted in different colors (yellow: TPP-binding module; green: PYR binding domain; blue: transketolase C-terminal domain).

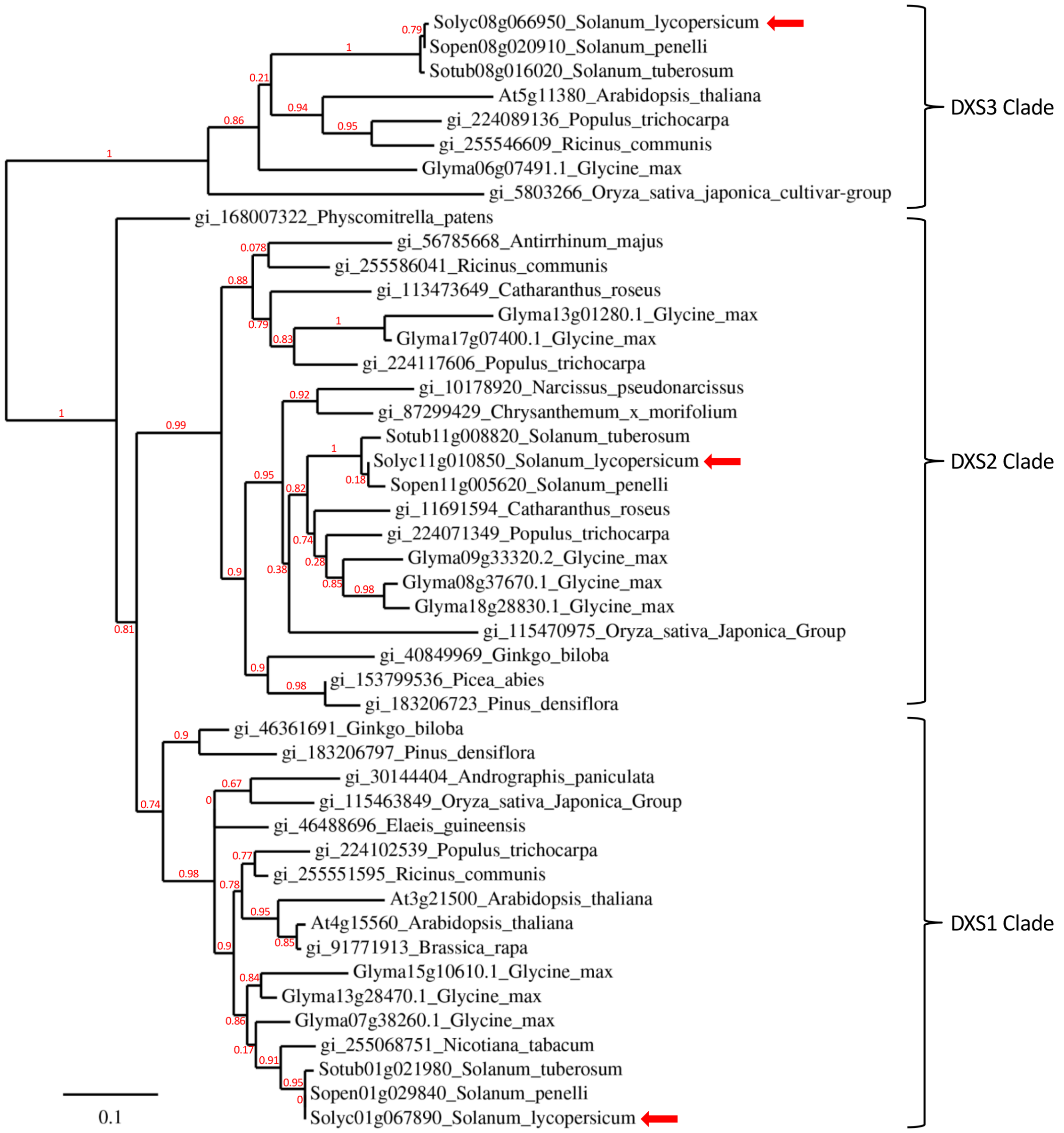

Figure S4. Phylogeny tree from 46 proteins included in the DXS family. Phylogenetic tree generated from aligning protein sequences of *S. lycopersicum* (arrows) against other bona fide plant DXS enzymes. The phylogenetic tree was constructed using Phylogeny.fr analysis (Dereeper et al., 2008) by maximum likelihood method with aLTR (approximate likelihood ratio). The branch support values are indicated above each branch.

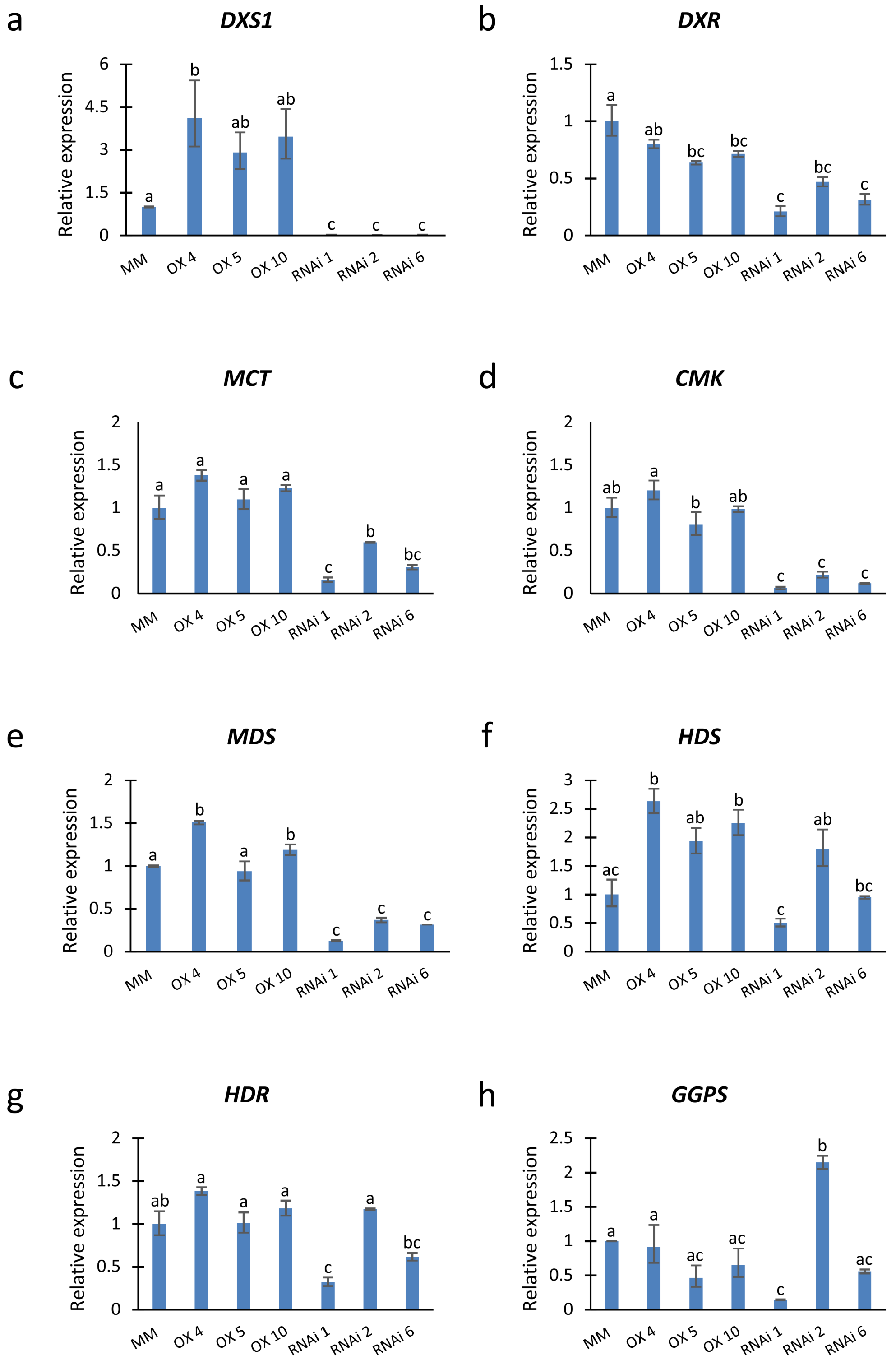

Figure S5. Influence of *DXS1* overexpression and silencing on the MEP pathway. Expression analysis of the MEP pathway genes, i.e. *DXS1* (a), *DXR* (b), *MCT* (c), *CMK* (d), *MDS* (e), *HDS* (f), *HDR* (g) and *GGPS* (h), in three *DXS1* transgenic lines showing either the highest or lowest *DXS1* expression levels. Error bars show the standard error value of two biological and two technical replicates. Values followed by the same letter (a, b, or c) are not statistically different (Fisher´s Least Significant Difference test, $P < 0.05$).

Table S1. Primer sequences used in this study.

A. Primers used for anchor-PCR Analysis

	Primer name	Primer sequence (5'-3')
	Adaptor-1	CTAATACGACTCACTATAGGC
	Adaptor-2	CTATAGGGCTCGAGCGGC
	Adaptor-3	AGCGGCGGGGAGGT
	Anchor Right-1	ACAGTTTTCGCGATCCAGAC
	Anchor Right-2	GGTCTTGCGAAGGATAGTGG
	Anchor Right-3	CTGGCGTAATAGCGAAGAGG
	Anchor Left-1	ATCGGTCTCAATGCAAAAGG
	Anchor Left-2	CGTCGAAATAAAGATTTCCGAAT
	Anchor Left-3	ATAATAACGCTGCGGACATCTAC

B. Primers used for genotyping analysis

	Primer name	Primer sequence (5'-3')
	Genotyping-F1	TTGCTGAATTACACGCCATT
	Genotyping-R1	TTTTTGGACAAATTACACAATTACA
	T-DNA-R1	CTGGCGTAATAGCGAAGAGG
	Genotyping-F2	GGCTTTCGTTTGATCAGAGATT
	Genotyping-R2	TTTTTGTCCTACGTGGTGTCAT
	T-DNA-F2	ATAATAACGCTGCGGACATCTAC

C. Primers used for amplified specific genes

Gene locus identity[a]	Primer name	Primer sequence (5'-3')
Solyc01g067860	S01g067860-F	TCGATGATCTCGTTGCGTTA
	S01g067860-R	TGAATGGCTCCCATTTTCTT
Solyc01g067870	S01g067870-F	TGGAGCAAAGTTGCACAAGA
	S01g067870-R	AAGAAACAGCATCACGAGCA
Solyc01g067880	S01g067880-F	TTTTCCTCCTTTGGACAAACA
	S01g067880-R	TCATGGTAGTGTAGTCGAAGCAA
Solyc01g067890	S01g067890-F	GCCATCAAGAAGCCCATCTA
	S01g067890-R	TGGCTTGTGAAGGCATTAAA

D. Primers used for generate transgenic plants

Gene locus identity[a]	Primer name	Primer sequence (5'-3')
Solyc01g067860	RNAi-POX-F	TCTAGACTCGAGCATTTGATAGCAATTATTTCAACATTC
Solyc01g067870	RNAi-POX-R	ATCGATGGTACCAATGGCTCCCATTTTCTTCA
Solyc01g067880		
Solyc01g067890	RNAi-DXS1-F	TCTAGACTCGAGCCTTCACATTCCTCCACATTG
	RNAi-DXS1-R	ATCGATGGTACCTCCAGAGAACAAAGGGGTTG
	35S-DXS1-F	GGATCCCACCAACACACCCCACTAGA
	35S-DXS1-R	GGTACCCAGCCATGGAAACTTTAAACA

Table S1. Continued.

E. Primers used for qRT-PCR Analysis		
Gene locus identity[a]	Primer name	Primer sequence (5'-3')
Solyc01g056940	LE-Ubi-F	CCAAGATCCAGGACAAGGAA
	LE-Ubi-R	AAATCAAACGCTGCTGGTCT
Solyc07g055920	ALQ-Pro-F	GGGAAGGTTCCCTACAATG
	ALQ-Pro-R	GGGGTTGCTCATCCATTA
Solyc01g067890	DXS1-F	TTCTTCAAACATGCATAACAAAGTT
	DXS1-R	CGAGTCGAAGTCGAGATTTG
Solyc11g010850	DXS2-F	TGGATTTAACTGTGGCTTTGC
	DXS2-R	TCCTGTCAAGATTTTGTGTGC
Solyc08g066950	DXS3-F	CCGGAGGTTTAGAGAACTTGC
	DXS3-R	CCAATCATACCACGTGCATAC
Solyc01g067860	S01g067860 Fz	CTAATTAGGCTTAAGTCCTTCAC
	S01g067860 Rz	CAGCAACTCCACCAAGAACA
Solyc01g067870	S01g067870 Fz	GAGTACTTGGGGTGCAAGGA
	S01g067870 Rz	TTGGACAATGAGTGCTTTGA
Solyc01g067880	S01g067880 Fz	GGGTGCTAGATTGCTTCGAC
	S01g067880 Rz	TGGTACAGCCTCCTTCTCTG
Solyc03g114340	DXR-Fz	TTGCTTTCTCCTGCTGAAAT
	DXR-Rz	CCCACTGCACTCCTTTCTTC
Solyc01g102820	MCT-Fz	ACCCAAGAAGGGGTCTTTTC
	MCT-Rz	TTCCTCCAGCAAGAAGAATCA
Solyc01g009010	CMK-Fz	GAAGGCTTCAGGTTCAAGAACTA
	CMK-Rz	AAGCCCAGCTTCTCTATCCA
Solyc08g081570	MDS-Fz	CGATTGGAACCTGGTTATCCT
	MDS-Rz	TCCCAAAATTGCATCAACAA
Solyc11g069380	HDS-Fz	TGAACTTCAGCCTGCATCC
	HDS-Rz	ACGTTCCCAACCATCACAGT
Solyc01g109300	HDR-Fz	AAGGTTTTGGACACAAAGAAGC
	HDR-Rz	CGGTTACGTTTCCCCATGTA
Solyc11g011240	GGPS-Fz	CTCTGCATGGCTTCTTGTGA
	GGPS-Rz	TCATGGACGAGTGACATGGT
